# Partners in Leaky Gut Syndrome: Intestinal Dysbiosis and Autoimmunity

**DOI:** 10.3389/fimmu.2021.673708

**Published:** 2021-04-22

**Authors:** Yusuke Kinashi, Koji Hase

**Affiliations:** ^1^ Division of Biochemistry, Faculty of Pharmacy and Graduate School of Pharmaceutical Science, Keio University, Tokyo, Japan; ^2^ International Research and Developmental Center for Mucosal Vaccines, The Institute of Medical Science, The University of Tokyo, Tokyo, Japan

**Keywords:** epithelial barrier, leaky gut syndrome, microbiota, dysbiosis, gut immune system, autoimmune diseases

## Abstract

The intestinal surface is constitutively exposed to diverse antigens, such as food antigens, food-borne pathogens, and commensal microbes. Intestinal epithelial cells have developed unique barrier functions that prevent the translocation of potentially hostile antigens into the body. Disruption of the epithelial barrier increases intestinal permeability, resulting in leaky gut syndrome (LGS). Clinical reports have suggested that LGS contributes to autoimmune diseases such as type 1 diabetes, multiple sclerosis, rheumatoid arthritis, and celiac disease. Furthermore, the gut commensal microbiota plays a critical role in regulating host immunity; abnormalities of the microbial community, known as dysbiosis, are observed in patients with autoimmune diseases. However, the pathological links among intestinal dysbiosis, LGS, and autoimmune diseases have not been fully elucidated. This review discusses the current understanding of how commensal microbiota contributes to the pathogenesis of autoimmune diseases by modifying the epithelial barrier.

## Introduction

The intestinal mucosa is exposed to a myriad of external antigens such as food antigens, food-borne pathogens, and commensal microbes that reside in the intestinal lumen. Therefore, the intestine serves as a barrier tissue whereby a monolayer of intestinal epithelial cells establishes a multilayered physicochemical barrier (1). The intestinal epithelial barrier contributes to the maintenance of biological homeostasis by segregating the internal and external milieus by restricting the infiltration of external antigens and the leakage of endogenous substances. To this end, intestinal epithelial cells form tight junctions (TJs) (2). TJ protein complexes tightly connect epithelial cells to reduce paracellular permeability. The main integral proteins of the TJs include occludin and claudins (3, 4). Their intracellular domains are associated with zonula occludens (ZO) proteins that connect the junctional complexes with myosin 1C, an important component of the actin cytoskeleton (5, 6). Furthermore, myosin light chain kinase (MLCK) acts with peri-junctional actomyosin rings to regulate the contractility of actin fibers, thereby influencing TJ structure and permeability (7, 8).

The mucosal barrier also includes mucin, antimicrobial peptides, and dimeric (or more polymeric) IgA secreted by goblet cells, Paneth cells, and plasma cells, respectively (9–11). These effector molecules constitute a barrier between luminal microbes and intestinal epithelium to prevent microbial adherence to the epithelium. However, mucosal barrier dysfunction (especially the disruption of TJs) often leads to enhanced intestinal permeability (12), a pathological status termed “leaky gut syndrome” (LGS). LGS initiates inflammatory responses in the intestine and in extraintestinal tissue (13, 14). Thus, the translocation of commensal microbes into the body disturbs immune homeostasis by inducing systemic inflammation; however, the commensal microbiota is important for shaping the gut immune system while they remain confined in the intestinal lumen (15). Such beneficial effects are ascribed to certain microbial products that promote the proliferation and differentiation of intestinal epithelial cells and multiple immune cell subsets including regulatory T cells and T helper type 17 (Th17) cells (16). Indeed, germ-free mice exhibit defects in the maturation of gut-associated lymphoid tissues and mesenteric lymph nodes, leading to attenuated production of secretory IgA (S-IgA) (17).

Altered microbial composition, termed dysbiosis, has been implicated in mucosal barrier dysfunction and inflammatory responses, which predispose the host animals to systemic diseases (e.g., inflammatory bowel disease, celiac disease, food allergy, obesity, and autoimmune diseases) (18). Accumulating reports have revealed that both LGS and dysbiosis are evident in some patients with autoimmune diseases (**Table 1**). In humans, lactulose/mannitol or lactulose/rhamnose tests have been used to assess intestinal permeability by measuring the urinary excretion of unabsorbed lactulose and absorbed mannitol or rhamnose. The lactulose/mannitol or lactulose/rhamnose ratio increases in patients with multiple sclerosis, rheumatoid arthritis, type 1 diabetes, or celiac disease (19, 24, 27, 30). Moreover, serum concentrations of lipopolysaccharide and soluble CD14 are indicators of intestinal permeability. Elevated serum lipopolysaccharide concentration and reduced TJ-related protein concentrations are observed in patients with ankylosing spondylitis or autoimmune hepatitis (22, 26). Likewise, serum soluble CD14 concentrations are elevated in those with systemic lupus erythematosus (32). These patients with autoimmune disease exhibit altered microbial compositions, compared with healthy volunteers (20, 23, 25, 26, 28, 31, 33). Thus far, it remains uncertain whether LGS and dysbiosis are causes or consequences of autoimmune diseases.

**Table 1:** Autoimmune diseases related to LGS and dysbiosis.

Pathological site	Disease name	Symptoms of LGS^a^	Characterization of dysbiosis^b^	Reference
Central nervous system	Multiple sclerosis	serum zonulin↑ lactulose/mannitol ratio↑	Methanobrevibacter, Akkermansia↑ Butyricimonas↓	(19–21)
Spinal cord	Ankylosing spondylitis	serum LPS, ileal zonulin↑ ileal TJ-related proteins↓	Prevotella↑ Bacteroides↓	(22, 23)
Joint	Rheumatoid arthritis	serum zonulin↑ lactulose/mannitol ratio↑	Prevotella↑ Bacteroides↓	(24, 25)
Liver	Autoimmune hepatitis	plasma LPS↑ duodenal TJ-related proteins↓	aerobic bacteria↑ anaerobic bacteria↓	(26)
Pancreas	Type 1 diabetes	serum zonulin↑ lactulose/rhamnose ratio↑	Bacteroides↑ short chain fatty acids-producing bacteria↓	(27–29)
Small intetine	Celiac disease	lactulose/mannitol ratio↑	Enterobacteriaceae, Staphylococcaceae↑ Streptococcaceae↓	(30, 31)
Systemic	Systemic lupus erythematosus	serum soluble CD14↑	Firmicutes/Bacteroidetes ratio↓	(32, 33)

a Upward and downward arrows represent an increase and decrease in the biological markers, respectively.

b Upward and downward arrows represent over-representation and under-representation of the indicated bacteria at the phylum, family or geneus level, respectively.

Early research has shown that proinflammatory cytokines (e.g., tumor necrosis factor-α [TNF-α] and interferon-γ) impair TJ integrity (34–36), whereas immunosuppressive cytokines (e.g., interleukin [IL]-10 and transforming growth factor-β) reinforce the TJs (37, 38). IL-22 secreted by intestinal immune cells is also vital for epithelial homeostasis (i.e., epithelial repair and intestinal stemness) as well as epithelial barrier functions (39). In support of this view, IL-22 induces claudin-2 to facilitate the clearance of enteric pathogens under physiological conditions (40). However, under inflammatory conditions such as Crohn’s disease, constitutive expression of claudin-2 by IL-22 eventually leads to an increment of intestinal permeability (41, 42). Thus, the cytokine milieu is a critical factor that influences epithelial barrier function. Given that the gut commensal microbiota plays an essential role in regulating gut immunity, the microbiota should affect the epithelial barrier by regulating cytokine-induced barrier changes. In this review, we discuss the link between the commensal microbiota and epithelial barrier function, as well as the potential contribution of dysbiosis-associated LGS to the pathogenesis of autoimmune diseases.

## Regulatory Mechanisms of the TJ Barrier

The innate immune system can sense pathogen-associated molecular patterns *via* pattern recognition receptors, including Toll-like receptors (TLRs) and nucleotide-binding oligomerization domain-containing proteins (i.e., NOD1 and NOD2) (43). Intestinal epithelial cells also express most TLRs and both NODs, among which TLR2 and TLR4 signaling may influence the integrity of TJ complexes. TLR2 recognizes lipopeptides, which are major cell wall components of bacteria. TLR2 signaling activates protein kinase C (PKC) and consolidates junctional complexes by recruiting ZO-1 *in vitro (* 44). In contrast, TLR4 signaling (mediated by myeloid differentiation primary response 88 [MyD88]) enhances intestinal permeability, both *in vitro* and *in vivo* (45). TLR4 signaling activates MLCK by initiating the canonical nuclear factor-κB pathway (46, 47), leading to cytoskeletal contraction that relaxes the TJ barrier. Thus, the epithelial sensing of various pathogen-associated molecular patterns by pattern recognition receptors positively or negatively regulates intestinal permeability at TJs.

Endogenous machinery to suppress TJs is regulated by zonulin, a eukaryotic analog of the ZO toxin produced by *Vibrio cholerae* (48). In humans, zonulin was identified as pre-haptoglobin (preHp)-2, the precursor of haptoglobin, which is enzymatically cleaved into the mature protein (49). Zonulin was released when mammalian small intestinal tissues were cocultured with pathogenic and nonpathogenic bacteria *ex vivo* (50). This observation suggested that bacterial exposure is a critical inducer of zonulin, although the underlying mechanism remains unclear. Furthermore, gliadin (a glycoprotein present in wheat)-dependent zonulin release is well-documented, especially in studies of celiac disease. Gliadin binds to the chemokine receptor CXCR3 (expressed by intestinal epithelial cells) to facilitate zonulin secretion in the MyD88-dependent pathway (51). Zonulin possesses epidermal growth factor (EGF)-like and proteinase-activated receptor 2 (PAR2)-activating peptide-like motifs; thus, it serves as a ligand for EGF receptor (EGFR) and PAR2 on intestinal epithelial cells (49). Zonulin-dependent activation of PAR2 reinforces EGFR signaling, which further activates PKC and leads to the phosphorylation of ZO-1 and myosin 1C (52). This sequence of events disrupts the associations of ZO-1 with the other TJ molecules and myosin 1C. Activated PKC also phosphorylates G-actin and causes actin polymerization (53). These effects of PKC activation synergistically promote TJ disassembly and enhance intestinal permeability. However, considering that TLR2 signaling-dependent activation of PKC recruits ZO-1 to TJs, the effect of PKC on TJ assembly remains controversial and may depend on the targets of phosphorylation. Furthermore, EGFR activation by EGF in the breast milk inhibits TLR4 signaling to protect neonates and infants from necrotizing enterocolitis (21). Thus, the effect of EGFR signaling on epithelial barrier functions may be context-dependent. In patients with celiac disease, the expression of CXCR3 is upregulated in the small intestine, including the epithelium (51). This event may enhance zonulin secretion, thereby causing barrier dysfunction and an inflammatory response to gluten.

The biological impact of zonulin on the intestinal epithelial barrier and the immune system has been defined in studies of zonulin-overexpressing mice, in which the mouse *Hp1* gene is replaced with the human *Hp2* (h*Hp2*) gene (29). Consequently, h*Hp2* knock-in enhanced intestinal permeability and promoted the development of dextran sodium sulfate (DSS)-induced colitis (54). h*Hp2* knock-in mice also exhibited a proinflammatory immune response mediated by RORγt^+^ cells, especially IL7R^+^ CD3^-^ RORγt^+^ (most likely, group 3) innate lymphoid cells in the small intestine (55). These data illustrate that zonulin overexpression may be implicated in the pathogenesis of chronic inflammatory diseases, including inflammatory bowel disease, and autoimmune diseases (56). Indeed, the serum concentrations of zonulin were significantly elevated in patients with multiple sclerosis, ankylosing spondylitis, rheumatoid arthritis and type 1 diabetes compared with those concentrations in healthy volunteers (22, 24, 57, 58). Furthermore, enhanced intestinal permeability, combined with the upregulation of zonulin and downregulation of TJ-related proteins, was evident in mice with collagen-induced arthritis, a model of rheumatoid arthritis (24). Importantly, these pathological events were observed before the onset of arthritis; treatment with a zonulin antagonist, larazotide, ameliorated the disease symptoms by improving barrier function. Thus, LGS mediated by zonulin most likely contributes to the development of collagen-induced arthritis. In human clinical trials, larazotide acetate also improved the symptoms in patients with celiac disease (59). Taken together, these observations support the importance of zonulin as a biomarker of intestinal permeability and a promising therapeutic target for LGS-associated autoimmune diseases (**Table 1**). Nevertheless, recent reports have shown that zonulin is inappropriate as a biomarker for irritative bowel syndrome, functional dyspepsia and non-Coeliac wheat sensitivity (60). There was only a weak correlation between zonulin level and intestinal permeability (61). This could be due to the detection method; widely distributed ELISA for zonulin measurement fails to quantify zonulin levels correctly. It is, therefore, paramount to establish the precise measurement system and to further investigate the causal relationship of zonulin and LGS-associated diseases using animal models like h*HP2* knock-in mice.

## Barrier Maintenance by Microbial Products

The commensal microbiota produces a considerable amount of various fermentation products (62), such as short-chain fatty acids (derived from dietary fibers and mucin glycans) (63), indoles (derived from tryptophan), and hydroxy fatty acids (derived from unsaturated long-chain fatty acids). Therefore, the commensal microbiota is often regarded as “a hidden organ.” Commensal microbiota-derived metabolites have substantial impacts on host physiological functions through metabolic reprograming (64), epigenetic modifications (65), and the activation of specific receptors like G protein-coupled receptors (GPRs) and aryl hydrocarbon receptor (AhR). There is increasing evidence that microbial metabolites can serve as exogenous regulators for the TJ barrier. For instance, butyrate, a short-chain fatty acid, augments the TJ barrier by inducing the hypoxia response. Colonocytes actively utilize butyrate as a critical energy source *via* beta-oxidation and subsequent oxidative phosphorylation. This metabolic process, which requires oxygen consumption, contributes to the establishment of anaerobic conditions in the colonic lumen and results in the stabilization of hypoxia-inducible factor-1 (HIF-1) in colonocytes (66). Consequently, butyrate upregulates *Cldn1* (encoding Claudin-1) and *Ocln* (encoding occludin) in a HIF-1-dependent manner, thereby conferring resistance to barrier disruption and bacterial translocation upon infection with *Clostridium difficile* (67).

Microbial indoles also regulate the integrity of TJs. In intestinal epithelial cells, indole-3-propionic acid downregulates TNF-α and upregulates TJ-related proteins in a pregnane X receptor (PXR)-dependent manner (68). PXR-deficient mice exhibit an LGS-like phenotype and high susceptibility to indomethacin-induced enteritis. Because PXR/TLR4-double deficiency rescues the LGS-like phenotype, indole-3-propionic acid presumably counteracts TLR4-mediated barrier dysfunction. Additionally, oral administration of indole-3-ethanol, indole-3-pyruvate, and indole-3-aldehyde mitigates DSS-induced colitis by securing the TJ barrier in an AhR-dependent manner (69). AhR signaling downregulates the expression of MLCK, which results in the dephosphorylation (and subsequent activation) of non-muscle myosin II-A and ezrin under inflammatory conditions. Importantly, both myosin II-A and ezrin are TJ-associated actin regulatory proteins that can destabilize TJ complexes (70, 71).

Urolithin A (derived from polyphenols) also acts as a TJ modulator through AhR signaling (72). Urolithin A-dependent activation of AhR upregulates the expression levels of *Cldn4*, *Ocln*, and *ZO-1* by inducing Cyp1A1 and Nrf2. The administration of urolithin A mitigates barrier dysfunction and colitis development in the mouse model of 2,4,6-trinitrobenzene sulfonic acid-induced colitis; this protective effect is attenuated in mice lacking either Nrf2 or AhR. These findings imply that urolithin A requires both AhR- and Nrf2-dependent pathways to enhance the TJ barrier.

Gut-resident *Lactobacillus* spp. produces unique hydroxy fatty acids such as 10-hydroxy-*cis*-12-octadecenoic acid (HYA) (73). HYA binds to GPR40 on Caco-2 intestinal epithelial cells to activate the mitogen-activated protein kinase/extracellular-signal-regulated kinase pathway, thereby upregulating TJ-related proteins (74). Treatment with HYA was protective against IFN-γ and TNF-α-induced barrier disruption *in vitro* and the development of DSS-induced colitis *in vivo*. Furthermore, HYA considerably enhances the fecal IgA concentration in the NC/nga mouse model of atopic dermatitis (75), indicating that the protective effect of HYA on the colitis model may be attributed to the reinforcement of an epithelial barrier and an augmented S-IgA response.

Multiple lines of investigation have suggested that epithelial barrier dysfunction may result from the loss of beneficial species due to intestinal dysbiosis. In *db/db* mice that spontaneously develop type 2 diabetes, epithelial dysfunction is accompanied by underrepresentation of the major butyrate producer, *Faecalibacterium prausnitzii* (76). *F. prausnitzii* is also nearly absent from Crohn’s disease-associated gut microbiota (63, 77). Importantly, *F. prausnitzii* produces microbial anti-inflammatory molecule, which consolidates TJ integrity by upregulating *ZO-1.* Treatment of *db/db* mice with the *F. prausnitzii*-derived anti-inflammatory molecule restored *ZO-1* expression and improved intestinal permeability. Additionally, the outer membrane protein of *Akkermansia muciniphila*, Amuc_1000*, upregulates *Cldn3* and *Ocln* at least partially through the activation of TLR2 signaling (78). High-fat diet (HFD)-induced obesity is associated with a lower abundance of *A. muciniphila*, while the administration of Amuc_1000* reduces body fat mass by alleviating HFD-induced endotoxemia. Notably, *A. muciniphila* is regarded as a mucin-degrading species, which may affect the mucin barrier (79, 80). Taken together, these observations imply that specific symbionts shape epithelial barrier function by providing beneficial metabolites and proteins.

## Barrier Disruption by Specific Microbes

**Figure 1:**
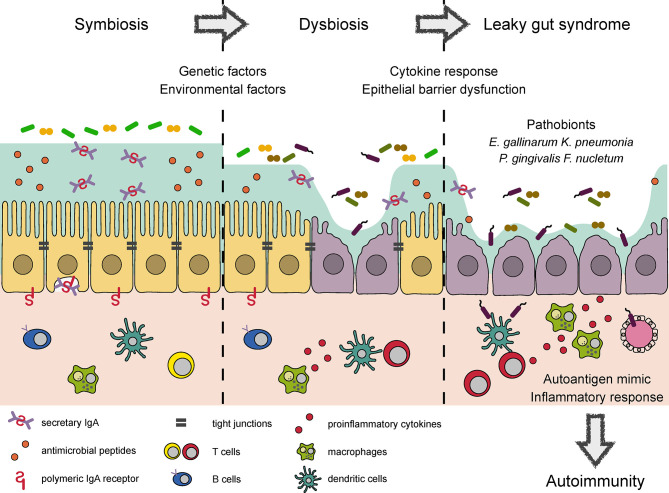
Conceptual diagram of autoimmune responses induced by dysbiosis and LGS. Several bacterial products reinforce epithelial barrier and regulate the mucosal immune response to maintain symbiotic relationship in the intestine. Environmental factors such as a westernized diet and drugs cause dysbiosis, which impairs epithelial barrier function and elicits proinflammatory response. Microbial adhesion to epithelial cells and the induction of proinflammatory cytokines further damage TJ integrity, leading to LGS. LGS enhances bacterial translocation to the systemic circulation. Some of the translocated bacteria provide mimotopes or serve as adjuvants to initiate or worsen autoimmune responses, respectively.

Intestinal pathobionts are often overrepresented in the microbiota of patients with inflammatory disorders, where they accelerate systemic inflammation by translocating across the epithelial barrier to reach extraintestinal tissue (**Figure 1**). A notable example of such pathobionts is *Enterococcus gallinarum*, which is frequently detected in the livers of patients with systemic lupus erythematosus and autoimmune hepatitis (81). In systemic lupus erythematosus model (NZW × BXSB F1 hybrid) mice, colonization by *E. gallinarum* caused barrier dysfunction and bacterial translocation to the liver, thereby exacerbating autoantibody production through the upregulation of hepatic autoantigen expression. The monoassociation of *E. gallinarum* in germ-free mice also recapitulated an LGS-like phenotype with enhanced bacterial translocation to the liver, presumably due to the induction of *Hp/zonulin* and the reciprocal downregulation of TJ-related molecules (e.g., *Cldn3* and *Ocln*).

Patients with primary sclerosing cholangitis (PSC) possess several bacterial species with barrier-disrupting property (82). More than 70% of patients with PSC exhibit comorbid ulcerative colitis (UC). Fecal microbiota transplantation from PSC-UC patients to germ-free mice provoked systemic translocation of *E. gallinarum, Proteus mirabilis*, and *Klebsiella pneumonia.* Among these species, *K. pneumonia* can damage epithelial cells, leading to enhanced intestinal permeability. Eventually, colonization by the PSC-UC microbiota or a mixture of the three bacterial strains exacerbated of 3,5-dicarbethoxyl-1,4-dihydrocollidine-induced hepatobiliary injury by activating hepatic Th17 response.

There is compelling evidence for a link between oral and gut microbiota. In particular, oral dysbiosis and proton pump inhibitor usage facilitate the translocation of otherwise oral-indigenous bacteria to the intestine (83). Importantly, *Porphyromonas gingivalis*, a periodontopathic bacterium, may predispose hosts to systemic inflammation and autoimmunity by inducing LGS. In support of this view, the administration of *P. gingivalis* has been shown to alter the gut microbial composition and suppress the expression of TJ-related proteins, thereby augmenting the systemic translocation of bacteria and their products (84, 85). The administration of *P. gingivalis* accelerates metabolic syndrome, collagen-induced arthritis, and experimental autoimmune encephalomyelitis (EAE) (84, 86, 87). Another oral microbe, *Fusobacterium nucleatum*, also induces intestinal dysbiosis and LGS by suppressing the expression of both ZO-1 and occluding. Therefore, *F. nucleatum*-treated mice are highly susceptible to DSS-induced colitis (88). Notably, *F. nucleatum* is often detected in patients with colorectal carcinoma (89, 90). Based on these data, specific oral pathobionts presumably play vital roles in the development of inflammatory disorders through LGS (**Figure 1**). However, the underlying mechanism by which oral pathobionts disrupt the gut microbial community remains to be elucidated.

## Pathological Contribution of Dysbiosis and LGS to Autoimmune Diseases

Exogenous (e.g., diet and drugs) and endogenous factors (e.g., antimicrobial peptides, S-IgA, and the mucin layer) are known to affect the gut microbial community. For instance, a HFD reduces the abundance of *Bacteroidetes* and reciprocally enhances the abundances of *Firmicutes* and *Proteobacteria* (91). Low-fiber diet and high-glucose intake enhance the proportions of mucin-degrading bacteria (79, 80). These findings suggest that a westernized diet affects the microbial community. Antibiotics is another major contributor to alter microbial composition (92); as mentioned above, proton pump inhibitors also promote the translocation of oral pathobionts to the intestine (83).

In addition, mutations in several genes (i.e., *NOD2* and *XBP1*) and the presence of environmental stress (e.g., obesity and irradiation) causes Paneth cell dysfunction, which impairs the secretion of antimicrobial peptides and causes dysbiosis (93). Furthermore, patients with selective IgA deficiency who have serum IgA concentrations of < 7 mg/dL exhibit intestinal dysbiosis and high susceptibility to allergic and autoimmune diseases (e.g., type 1 diabetes, rheumatoid arthritis, and systemic lupus erythematosus) (94–96). *Sutterella* spp. are known to possess S-IgA-degrading activity (97). Colonization with *Sutterella* spp. enhances susceptibility to DSS-induced colitis by reducing the amount of luminal S-IgA.

Polarized protein sorting abnormalities cause barrier dysfunction and dysbiosis. In polarized epithelium, adaptor protein-1B (AP-1B) complex mediates clathrin-dependent polarized protein sorting (98). We previously showed that a deficiency of *Ap1m2* (encoding the μ1B subunit of AP-1B complex) interferes with the basolateral sorting of several cytokine receptors (e.g., IL-6st, IL-17RA, tumor necrosis factor-RII, and transforming growth factor-βRI) (99). These abnormalities attenuate cytokine signaling and downregulate the expression of antimicrobial peptides in the intestinal epithelium. *Ap1m2* deficiency also disturbs IgA transcytosis to the intestinal lumen due to the inappropriate sorting of polymeric immunoglobulin receptor. Consequently, *Ap1m2-*deficient mice exhibit dysbiosis and LGS, leading to the spontaneous development of Th17-mediated chronic colitis. The importance of AP-1B-mediated maintenance of epithelial integrity in systemic immune homeostasis is currently under investigation.

Microbial adhesion to the epithelium could initiate a sequence of inflammatory responses by activating signal transduction *via* TLRs and zonulin signaling, leading to the loss of TJ integrity. Such chronic barrier dysfunction causes bacterial translocation and an inflammatory response that further damages the TJ barrier and also induce epithelium apoptosis by inflammatory cytokines (100). This vicious cycle potentiates the autoimmune response in genetically susceptible patients and may trigger an acquired autoimmune response even in genetically normal individuals. Indeed, an experimental observation has verified that LGS promotes genetically induced autoimmunity. Induction of LGS by DSS administration leads to the activation of autoreactive T cells in the intestine of type 1 diabetes model NOD mice carrying an islet-reactive T cell receptor (101). Eventually, this response elicits diabetes; however, antibiotics treatment canceled the disease development.

Accumulating evidence implies that cross-reactivity to microbial antigens may trigger autoimmune responses. Common microbial peptides (GTP-binding protein engA) with homology to myelin basic protein induce the antigen-specific T cell response by low-affinity T cell recognition (102). In this study, the humanized mice carrying HLA-DR2 haplotype (DRB1*1501) and myelin basic protein-specific human T cell receptor developed multiple sclerosis-like symptoms upon immunization with the microbial peptides. Besides, *P. gingivalis* may act as a mimic antigen to induce autoimmunity. It is well documented that patients with rheumatoid arthritis possess antibodies against anti-citrullinated proteins such as α-enolase, contributing to the pathogenesis (103). Human α-enolase shares homology with *P. gingivalis*-derived α-enolase, and thereby the human citrullinated α-enolase-specific antibodies cross-reacts with citrullinated *P. gingivalis* α-enolase (104). Ruff et al. recently demonstrated that the DNA methyltransferase of *Roseburia intestinalis*, a major commensal species, also serves as a mimotope of human β_2_-glycoprotein I in patients with antiphospholipid syndrome (105). This mimotope presumably facilitates the generation of autoreactive Th1 cells and autoantibodies. Administration of *R. intestinalis* to NZW × BXSB F1 hybrid mice causes antiphospholipid syndrome-like symptoms by inducing autoimmunity to β_2_-glycoprotein I. Miyauchi et al. also revealed that The UvrABC system protein A (UvrA) expressed by *Lactobacillus reuteri* is a mimotope of mouse myelin oligodendrocyte glycoprotein, an antigen used to induce EAE (106). Monoassociation by *L. reuteri* alone moderately promotes EAE progression; however, co-association with *Erysipelotrichaceae* possessing an epithelium-attaching property markedly worsens disease progression.

Segmented filamentous bacteria (SFB) also attaches to the ileal epithelium to elicit the intestinal immune response such as Th17 response. This effect is mediated by serum amyloid A and reactive oxygen species by epithelial cells (107). Antigen presentation of SFB-derived antigens by intestinal dendritic cells is also required to induce Th17 cells (108). Accordingly, colonization by SFB facilitated the development of EAE due to enhanced Th17 response (109). Meanwhile, the SFB-dependent Th17 response suppressed the bacterial translocation in constitutively MLCK-activated mice (110). Based on these observations, SFB could be a double-edged sword that consolidates the barrier integrity but augments the autoimmune response in a context-dependent manner.

Collectively, genetic and environmental factors affect the microbial composition, leading to epithelial barrier dysfunction directly and/or indirectly by means of inflammatory responses. These pathological changes enhance the systemic translocation of luminal bacteria, some of which provide mimotopes or augment autoimmune responses (**Figure 1**).

## Conclusion and Perspectives

The commensal microbiota has critical regulatory influences on epithelial barrier function. While the dysbiosis-mediated induction of LGS initiates an inflammatory response, some microbial products reinforce TJ integrity. Given that the commensal microbiota contributes to the development of LGS-associated autoimmune diseases, interventions targeting the microbiota are emerging as new therapeutic strategies to prevent or cure autoimmune diseases. Probiotics and fecal microbiota transplantation have been investigated in clinical trials for the treatment of type 1 diabetes, multiple sclerosis, and rheumatoid arthritis (111, 112). However, the pathological mechanism underlying LGS-dependent autoimmunity remains mostly unknown. Moreover, the precise location (e.g., proximal or distal intestine) where epithelial barrier dysfunction occurs initially has yet to be determined. Further investigations using LGS model animals are needed to elucidate the pathogenesis and provide proof-of-concept for promising therapies for autoimmune diseases.

## Author Contributions

YK wrote the manuscript and prepared the figure and table. KH critically revised the manuscript and obtained grants. All authors contributed to the article and approved the submitted version.

## Funding

This study was supported by grants from the Japan Society for the Promotion of Science (20H00509, 20H05876, and JPJSBP 120207405 to KH), AMED-Crest (20gm1010004h0105 and 20gm1310009h0001 and to KH), and The Naito Foundation (KH).

## Conflict of Interest

The authors declare that the research was conducted in the absence of any commercial or financial relationships that could be construed as a potential conflict of interest.
